# Cancer Stem Cells in Pancreatic Cancer

**DOI:** 10.3390/cancers2031629

**Published:** 2010-08-19

**Authors:** Qi Bao, Yue Zhao, Andrea Renner, Hanno Niess, Hendrik Seeliger, Karl-Walter Jauch, Christiane J. Bruns

**Affiliations:** Department of Surgery, Ludwig Maximilian University of Munich, Klinikum Grosshadern, Marchioninistr. 15, D-81377, Munich, Germany; E-Mails: qi.bao@med.uni-muenchen.de (Q.B.); yue.zhao@med.uni-muenchen.de (Y.Z.); andrea.renner@med.uni-muenchen.de (A.R.); hanno.niess@med.uni-muenchen.de (H.N.); hendrik.seeliger@med.uni-muenchen.de (H.S.); karl-walter.jauch@med.uni-muenchen.de (K.W.J.)

**Keywords:** cancer stem cells, pancreatic cancer, signaling pathways, metastasis, CSC-related therapy, microRNAs

## Abstract

Pancreatic cancer is an aggressive malignant solid tumor well-known by early metastasis, local invasion, resistance to standard chemo- and radiotherapy and poor prognosis. Increasing evidence indicates that pancreatic cancer is initiated and propagated by cancer stem cells (CSCs). Here we review the current research results regarding CSCs in pancreatic cancer and discuss the different markers identifying pancreatic CSCs. This review will focus on metastasis, microRNA regulation and anti-CSC therapy in pancreatic cancer.

## 1. Introduction

Stem cells are a subpopulation of cells that have the capability to differentiate into multiple cell types and maintain their self-renewal activity. The current consensus definition describes a cancer stem cell (CSC) as a cell within a tumor which is able to self-renew and to produce heterogeneous lineages of cancer cells that comprise the tumor. Alternative terms in the literature for CSCs are “tumor-initiating cells” or “tumorigenic cells”. CSCs divide asymmetrically, resulting in self-renewal of the tumor-initiating cells and production of daughter cells known as transient-amplifying cells without self-renewing capabilities that divide indefinitely to contribute to cancer progression [1]. However, the origin of CSCs is still under discussion. Genomic instability causing spontaneous transformation of non-tumorigenic stem cells into CSCs is one potential source; another potential origin for CSCs are tissue resident stem cells acquiring a malignant phenotype by accumulation of mutations.

The first identification of CSCs in a human tumor was by Dick’s group in 1994, they isolated leukemia stem cells with CD34 and CD38 [2,3]. The first breakthrough for CSCs in solid tumor systems was in breast cancer, where Al-Hajj analyzed breast cancer stem cells using CD44 and CD24 as markers [4]. Since then, CSCs have been identified in almost all human cancers including glioblastoma [5], lung cancer [6], colon cancer [7], liver cancer [8] and pancreatic cancer. Li *et al*. performed the initial experiments with pancreatic CSCs by isolating EpCAM^+^CD44^+^CD24^+^ cancer cells with high tumorigenic potential [9]. Hermann *et al*. used CD133 as a marker to isolate pancreatic cancer cells with a significantly higher tumorigenic potential, and CD133^+^CXCR4^+^ cancer cells with a significantly higher metastatic potential in addition [10]. Recently, Rasheed *et al*. identified ALDH^+^ pancreatic cancer cells with stem cell like tumorigenicity, clonogenic potential and characteristics of epithelial-mesenchymal transition (EMT) [11].

## 2. Material and Methods

We accessed with PubMed to search for the articles for our review. The articles chosen were published from 1997 until June 2010 and were found using the terms “cancer stem cells”, “tumor initiating cells”, “cancer stem-like cells” in combination with “pancreatic cancer”, “pancreatic ductal adenocarcinoma”, “stem cell marker”, “signal pathway”, “metastasis”, “microRNAs” and “therapy”. Relevant English-language articles were reviewed, as were their reference lists to identify further relevant articles.

Here we review the current research results of CSCs in pancreatic cancer and discuss the different markers identifying pancreatic CSCs. This review will focus on metastasis, microRNA regulation and anti-CSC therapy in pancreatic cancer.

## 3. Identification of Stem Cell Markers for Pancreatic CSCs

How to investigate the stemness of pancreatic cancer cells? At the very beginning, according to the experiences of hematopoietic and brain malignancies, researchers prefer to use well acknowledged markers such as EpCAM, CD44, CD24, CD133, and CXCR4. All markers identifying cancer stem cells are surface markers, definitely expressed on the normal stem cells at the same time and apparently changing during cancer stem cell development. This unstable characteristic is confirmed by investigators in colon cancer stem cell using CD133 as marker. Shmelkov *et al*. demonstrated that CD133^−^ cancer cells also have tumorigenic potential in NOD/SCID mice as compared to CD133^+^ cancer cells [12]. Rasheed *et al*. reported that ALDH (aldehyde dehydrogenase) could potentially be an endogenous marker for pancreatic CSCs [11]. ALDH is an endogenous enzyme that plays a key role in ethanol and retinal metabolism as well as the pathogenesis of pancreatic cancer. Rasheed *et al*. analyzed first the clonogenic growth potential *in vitro* and secondarily tumorigenesis *in vivo* to confirm the stemness of ALDH^+^ pancreatic cancer stem cells. Although there is not any obvious overlap between ALDH^+^ and CD44^+^CD24^+^ CSCs, both of them show stemness *in vitro* and *in vivo*. These results indicate that different markers identify different subpopulations of CSCs and presumably these subpopulations are responsible for the diverse processes of differentiation, development, and metastasis in CSCs. 

**Figure 1:**
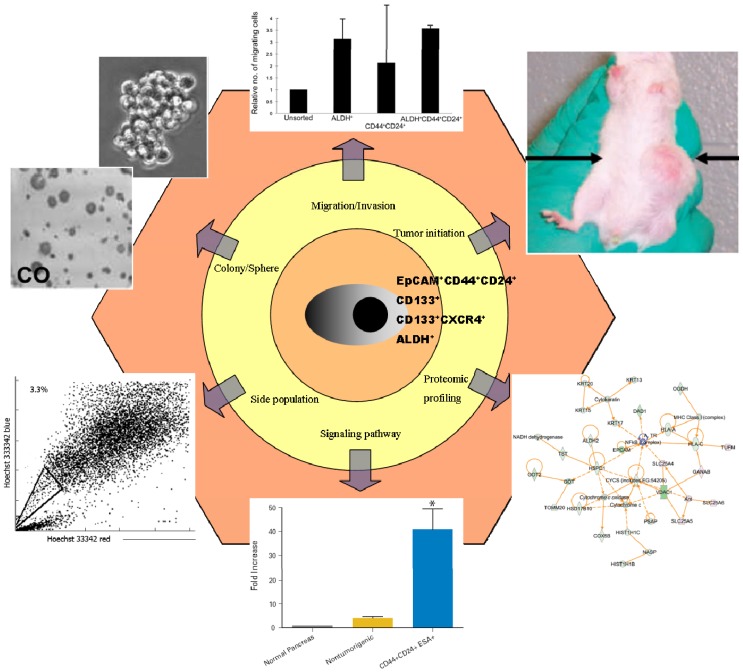
Identification of stemness in pancreatic cancer stem cells (CSCs). Pancreatic CSCs can be identified and sorted by surface markers such as EpCAM, CD44, CD24, CD133, CXCR4, and ALDH, as well as side population assays. Functional analysis either *in vivo* or *in vitro* should be performed to show the potential of tumor initiation, clonogenic growth metastases and/or invasion. Genomic and proteomic profiling assays help to analyze the RNA and protein expression and regulation. This figure was drawn according to references [9,11,13,14,15,16].

Recently, the functional analysis of CSCs aside of their surface marker profile appears to have moved into the spotlight of researchers’ interests. Dai and colleges performed a proteomic analysis of pancreatic CSCs [16]. Traditional signal pathway studies demonstrated activated stemness associated pathways such as SHH, BMI-1, BMP-4, *etc*. [9,10,17]. Rausch *et al*. studied the clonogenic growth potential of pancreatic CSCs by colony formation assay [15], and Rasheed analyzed the behavior of pancreatic CSCs in migration and invasion assays [11]. Furthermore, *in vitro* sphere formation assays were used to study the ability of tumor initiation [18] and pancreatic CSCs were serially transplanted into immunodeficient mice to show the tumorigenesis and stemness *in vivo* [11] (schematic chart is shown in Figure 1).

## 4. Signaling Pathways in Pancreatic CSCs

One of the most efficient, and certainly in part controversially discussed, strategies to delete the CSCs is to address therapy against particular CSC-associated signaling pathways such as the sonic hedgehog (SHH) pathway. Li *et al*. identified that SHH expression in CD44^+^CD24^+^ and EpCAM^+^ cancer cells is 46-fold higher than in normal pancreatic epithelial cells [9]. However, Yauch’s group showed that hedgehog ligands fail to activate signaling in tumor epithelial cells. Additionally Nolan-Stevaux’s data demosntrate that autocrine SHH signaling is not required in pancreatic cancer progression, but can be activated through ligand-dependence in the stromal microenvironment [19,20]. Interestingly, Feldmann *et al*. demonstrated compelling results in reduction of tumor invasion and metastasis by inhibition of the SHH signaling pathway accompanied with standard chemotherapy [21]. Recently, Jimeno *et al*. were able to show that chemotherapy resistant ALDH^+^ and CD24^+^ pancreatic CSCs can be eliminated by inhibition of the SHH signaling pathway with cyclopamine [22]. 

As investigated by Piccirillo *et al*. BMP-4 inhibits the tumorigenic potential of glioblastoma CSCs through the BMP-4/BMPR/SMAD signaling pathway [23]. Moreover, Hua *et al*. demonstrated that BMP-4 regulates the expansion and differentiation of pancreatic progenitor cells [17]. So, BMP-4 might also be related to pancreatic CSCs.

Interestingly, BMI-1, a member of the polycomb gene family, which is responsible for CSC self-renewal in leukemia, breast and brain cancer, is highly expressed in CD44^+^CD24^+^EpCAM^+^ pancreatic CSCs. BMI-1 is one of the activators in early stage pancreatic cancer and precursor lesions such as pancreatic intraepithelial neoplasia (PanIN) [14]. Some other upstream and downstream signaling molecules such snail, E-cadherin, and NF-κB [15,21], which are related to epithelial-mesenchymal transition (EMT) and metastasis play an important role in pancreatic CSCs. Hence, these signaling pathways could be promising targets for anti-CSC therapy combined with standard chemo- and radiation therapy. 

## 5. Involvement of Pancreatic CSCs in Metastasis

Metastasis is the predominant cause of cancer mortality. Pancreatic cancer is notoriously difficult to diagnose in early disease stages. At the time of diagnosis, 52% of patients suffer from distant metastases and 26% from locally advanced disease. Even after surgical resection of early diagnosed cases, most of these patients develop local recurrence and/or distant metastases [24]. The molecular mechanism of metastasis in pancreatic cancer is far from being clarified. Epithelial-mesenchymal transition (EMT) is required for tissue morphogenesis during embryonic development, comparably, EMT induction in cancer cells results in the acquisition of an invasive and metastatic phenotype [25]. Interestingly, a subpopulation of CD44 high/CD24 low CSC-like disseminated breast cancer cells were found in pleural effusions [4]. CD44 as a beta-catenin target gene plays a role in the EMT-associated Wnt signaling pathway for CSC maintenance [26,27]. As mentioned above, Li *et al*. identified a CD44^+^CD24^+^EpCAM^+^ subpopulation of tumor cells from primary human pancreatic cancer tissues and mice xenograft tumors. These CD44^+^/CD24^+^/EpCAM^+^ cells presented self renewal capacity, tumor initiation and up-regulated of the Hedgehog signaling pathway [9]. 

CD133 was used as CSC surface marker in various types of cancer, including brain, colon and pancreatic cancer [7,10,28,29,30]. Hermann *et al*. distinguished two subpopulations of CSC with individual phenotypes: CD133^+^CXCR4^+^ cancer cells as migrating CSCs with the properties to give rise to metastasis, and CD133^+^CXCR4^−^ CSCs without metastatic capacity. However, both of these subpopulations were able to induce primary tumor formation [10]. The involvement of CXCR4 as an alpha-chemokine receptor, specific for stromal-derived-factor-1 (SDF-1, also called CXCL12), has been repeated demonstrated for metastatic process in several solid tumor systems. The activation of the SDF-1-CXCR4 signaling pathway may promote the migration and adhesion of CXCR4^+^ disseminated cancer cells to endothelial and stromal ECM components at the distant metastatic sites [31,32,33]. Hence, CD133^+^CXCR4^+^ cancer cells might acquire a migratory phenotype during the EMT process and therefore are involved in invasion and metastases to distant sites [34]. 

Additionally, ALDH-positive tumor cells in primary pancreatic cancer tissues were associated with worse survival. As compared to the CD44^+^CD24^+^ CSC subpopulation, ALDH^+^CD44^+^CD24^+^ CSCs showed stronger tumor-initiating capacity, a significant mesenchymal gene profile and higher correlation to metastasis. Although primary tumors from six (75%) of the eight patients were ALDH negative, in four (67%) of these six cases the matched metastatic lesions contained ALDH-positive cells [11]. Mesenchymal features have been ascribed to invasive cells, and EMT related gene expression in cancers is highly associated with metastases [35].

## 6. MicroRNAs Regulate and Control EMT and Stem Cell Renewal

MicroRNAs (miRNAs) have emerged as essential regulators of differentiation and disease development [36]. Accumulated microarray data indicate different expression profiles of miRNAs in various types of human cancers [37,38]. Some of these small RNAs have been identified to be aberrantly expressed by CSCs and act as key regulators of CSC signaling [39]. It has been demonstrated that the miR-200 family members (miR-141, miR-200a, b, c and miR-429) are able to repress the expression of stem cell factors such as Sox2 and Klf4. Furthermore, Wellner *et al*. found a regulatory feedback loop between miRNAs, CSC and EMT in pancreatic cancer [40]. The function of ZEB1 as an important EMT-inducer and transcriptional repressor promoting invasion and metastasis [41] can be suppressed by miR-200 family members [42,43,44,45,46,47]. ZEB1 is expressed at the invasive front of pancreatic cancer and highly correlates with the expression of miR-200 family members and miR-203. The knock-down of ZEB1 resulted in an obvious epithelial transition in pancreatic cancer cell lines with mesenchymal phenotype (MiaPaCa2 and Panc1) and affected properties associated with CSCs including the incidence of CD24^+^ cancer cells, the ability to form spheres in culture, and development of chemoresistance. Another CSC-regulating miRNA complex in pancreatic cancer is the miR-34 family directly regulated by p53 and identified as tumor suppressor miRNAs in recent publications [48,49,50,51,52,53,54,55]. MiR-34a was used as a marker for invasiveness and metastasis in liver cancer [55]. Interestingly, miR-34 regulates Notch pathway proteins and Bcl-2; hence, miR-34 might play an important role in the maintenance and survival of CSCs. Ji *et al*. identified that CD44^+^CD133^+^ MiaPaCa2 cells are enriched with tumor sphere-forming and tumor-initiating cells or cancer stem/progenitor cells with high levels of Notch/Bcl-2 and a loss of miR-34. Recent data indicate that miR-34 restoration led to an 87% reduction of the tumor-initiating cell population accompanied by a significant inhibition of tumor cell sphere growth *in vitro* and tumor formation *in vivo* [56]. 

All above results suggest that CSCs might at least be in part responsible for the aggressive tumor biology in pancreatic cancer. Some biological key processes in CSCs seem to be regulated by miRNAs, which contributed positively to EMT for cancer metastasis. 

## 7. CSC-Related Therapy in Pancreatic Cancer

### 7.1. Resistance of CSCs to Current Therapy

CSCs seem to have activated cellular mechanisms allowing them to escape from standard chemo- or radiation therapy. In hematological malignancies, CSCs have been demonstrated to be responsible for therapy resistance and disease relapse.

Pancreatic cancer consists of different subpopulations of CSCs, which are resistant to standard chemo- or radiation therapy [57]. Hermann *et al*. discovered that gemcitabine as standard chemotherapy for pancreatic cancer did not induce apoptosis in the CD133^+^ subpopulation of the metastatic pancreatic cancer cell line L3.6pl as compared to the CD133^-^ control cells. Furthermore, increasing concentrations of gemcitabine treatment led to a significant enrichment of the CD133^+^ subpopulation in L3.6pl cells [10]. Similar results were obtained for the same CSC subpopulation using 5-FU (5-Fluorouracil) treatment [58]. Shah *et al*. generated gemcitabine resistant pancreatic cancer cells presenting with an over-expression of vimentin and a reduction E-cadherin expression in combination with the detection of a CD44^+^CD24^+^EpCAM^+^ subpopulation [59]. 

In addition, drug resistance is related to the cellar capacity of drug transportation. ATP-binding cassette (ABC) transporters like ABCG2 and ABCG5 have been shown to contribute to drug resistance in various types of cancers [60]. Another subpopulation of tumor cells named “side population”, mainly based on the presence and functionality of ABCG2, is currently estimated as a novel biomarker for CSCs. Olempska *et al*. identified ABCG2^+^CD133^+^ cells as individual CSCs, which represent resistance to chemotherapy as one of the CSC properties [61]. 

### 7.2. Targeting CSCs in Pancreatic Cancer

In the review of Hidalgo *et al*., the authors summarized commonly used treatment regiment and corresponding selected strategic targets in both early and advanced stage patients with pancreatic cancer [62]. It indicates that multiple application of traditional and novel agents (such as cytotoxic agent, monoclonal antibody, radioimmuno-conjugates, small-molecule inhibitor and even gene therapy), targeting diverse mechanisms of cancer, will help in establishing new approaches for pancreatic cancer therapy. Since CSCs are associated to therapy resistance and disease recurrence, the development of clinically useful inhibitors to target the individual CSC niche should be prioritized [63,64]. Furthermore, anti-CSC therapy should have minimal or no effect on normal stem cells. The therapy must be applied safely after testing for the sensitivity of normal and CSCs. It is probably one of the most visionary therapeutic strategies against solid tumor systems to combine an individualized anti-CSC approach by targeting their microenvironment or so called “CSC niche” with conventional radio- or chemotherapy for the remaining non CSC tumor cell proportion. 

### 7.3. MicroRNAs as a Class of Novel Therapeutic Tools for Pancreatic Cancer

MiRNAs can regulate the multiple processes involved in cancer initiation and metastases, some groups of miRNAs are known to influence CSC signaling and even CSC migration. Hence, miRNAs can be essential and efficient targets for tumor therapy. MiR-21 is an onco-miR implicated in esophageal, lung, stomach, liver, colon, and pancreatic cancer targeting tumor suppressor genes [65]. This important miRNA also emerged as a modulator regarding the response to chemotherapy. Antisense oligonucleotides against miR-21 generate a proapoptotic and antiproliferative response *in vitro* in different cellular models and reduce tumor development and metastatic potential *in vivo* [66]. Modified miRNA molecules with longer half-lives and better efficiency have been developed such as anti-miRNA oligonucleotides [67], locked nucleic acid (LNA) modified oligonucleotides [68] and cholesterol-conjugated antagomirs [69]. In addition, Ebert *et al*. have described a new approach to inhibit the function of miRNAs: synthetic mRNAs containing multiple binding sites for a specific miRNA, called miRNA sponges [70]. Take miR-10b therapeutic study as a case: miR-10b has been identified as one of the most significantly upregulated miRNAs in pancreatic cancer [71]. Ma and colleagues report the effects of systemic administration of antagomir-10b in a mouse breast cancer model. MiR-10b is induced by the transcription factor TWIST1 and targets HOXD10 mRNA. Orthotopic transplantation of 4T1 mouse mammary tumor cells (expressing high levels of TWIST1 and miR-10b) in syngeneic mice led to primary tumor growth with metastases to the lung. The authors found that systemic administration of antagomir-10b had marginal effects on the size and growth of the primary tumor, however, significantly reduced the number of metastases. They also reported that treatment with antagomir-10b did not suppress metastatic tumor growth when 4T1 cells were injected in the tail vein thereby bypassing the important steps of invasion and intravasation of the metastatic cascade of tumor cells. This study was thought as a phase I clinical trial of a targeted small interfering RNA drug—a new anti-metastasis agent—which is a new approach in anti-cancer therapy and potentially a future strategy to individually target CSCs [72].

## 8. Conclusions

Up until now, there are several markers related to pancreatic CSC such as EpCAM, CD44, CD24, CD133, and ALDH. However, among these markers, the overlap subpopulation of these marker-positive cells is too small to be isolated. 

Continuing identification of more specific surface or functional markers of CSCs, and better localization and visualization of different types of pancreatic cancer CSCs *in situ* potentially helps to facilitate the acquaintance with CSCs in the clinical routine with the major goal to develop an individualized therapeutic approach against these important subpopulations of pancreatic cancer cells. 

Finally, it appears clear to conclude that there is plenty of evidence for the existence of so-called “cancer stem cells” or “tumor-initiating cells” in pancreatic cancer, and that these cancer stem cells are at least in part responsible for aggressive local tumor growth and invasion, distant metastasis, and resistance to standard therapies. Additional therapeutic strategies targeting these subpopulations of cancer cells will improve the efficiency of standard chemo- and radiotherapy.
